# Lebanese *Cannabis sativa* L. extract protects from cisplatin-induced nephrotoxicity in mice by inhibiting podocytes apoptosis

**DOI:** 10.1186/s42238-025-00260-4

**Published:** 2025-01-16

**Authors:** Alia Khalil, Sahar Al Toufaily, Wassim Shebaby, Marissa El Hage, Dima Mroue, Wissam Faour, Mohamad Mroueh

**Affiliations:** 1 Gilbert and Rose-Marie Chagoury School of Medicine, Lebanese American University, P.O. Box36, Byblos, Lebanon; 2 School of Pharmacy, Pharmaceutical Sciences Department, Lebanese American University, Byblos, Lebanon; 3 Faculdade de Ciências Farmacêuticas, Universidade de São Paulo, São Paulo, Brazil; 4 Department of Natural Sciences, School of Arts and Sciences, Lebanese American University, Byblos, Lebanon

**Keywords:** *Cannabis sativa*, Cisplatin, Nephrotoxicity, Podocytes, Cisplatin-induced nephrotoxicity

## Abstract

**Background:**

Cisplatin is an anti-cancer drug used to treat a plethora of solid tumors. However, it is associated with dose dependent nephrotoxicity limiting its use as anticancer agent.

**Objective:**

The current study aimed to investigate the nephroprotective effect of native Lebanese *Cannabis sativa* in both in vitro and in vivo mice model of cisplatin-induced nephrotoxicity.

**Methods:**

Podocytes cell viability was assessed using MTS assay with cisplatin (30µM) in presence or absence of Cannabis oil extract (COE) at 0.5, 1 and 2µg/ml for 24h. Acute renal injury was established in adult female C57BL/6 mice with 20mg/kg, i.p. single dose cisplatin. Mice were divided into control group (vehicle), COE group, cisplatin group and cisplatin plus COE (2.5, 5 and 20mg/kg, i.p.). Animal body weight, serum creatinine, blood urea nitrogen (BUN), and proteinuria were measured.

**Results:**

Cell viability assay and western blot analysis revealed that COE prevented apoptosis induced by cisplatin in cultured immortalized rat podocytes. In addition, in vitro scratch assay demonstrated the ability of COE to promote and restore the migratory capacity of podocytes in cisplatin-treated cells.

Interestingly, COE treatment improved urinary and serum parameters characterized by a significant decrease in serum creatinine, urea, and proteinuria at various COE doses. Western blot analysis showed that COE inhibited COX-2 protein induction as well as apoptosis marker production (Bax/Bcl2 ratio) in cisplatin-treated mice when compared to mice treated with cisplatin alone.

**Conclusion:**

Collectively, the aforementioned findings indicate that COE could be a promising approach to protect against cisplatin-induced nephrotoxicity.

**Supplementary Information:**

The online version contains supplementary material available at 10.1186/s42238-025-00260-4.

## Introduction

Cisplatin, also known as diamminedichloridoplatinum (II), is a platinum compound and one of the most potent chemotherapeutic drug that has been approved by the FDA (Food and Drug Administration) in 1978 for the treatment of various solid malignancies including head and neck, testicular, ovarian, cervical cancer, bladder, melanoma, lymphomas and lung cancer (Aldossary 2019). Unfortunately, cisplatin chemotherapy is often associated with a cumulative and dose-dependent nephrotoxicity. Accordingly, 30% of patients treated with cisplatin develop renal dysfunction and acute kidney injuries (AKIs) few days following initial treatment (Pabla and Dong 2008).

After administration, cisplatin is actively transported across the plasma membranes via several transporters including *copper transporter-1 and 2 (CTR1 and CTR2)*, *P-type copper transporting ATPases (ATP7A and ATP7B)*, *organic cationtransporter-2 (OCT2)*, and *multidrug extrusion transporter- 1 (MATE1)*. While the expression of *CTR1*, *CTR2*, *ATP7A*, and *ATP7B* is ubiquitous, the kidneys express high levels of *OCTs* and *MATE1*that mediate a greater renal accumulation and toxicity of cisplatin (Ciarimboli 2012).

Although, renal damage through tubular cell death is the most commonly used model of cisplatin nephrotoxicity, the latter is a complex mechanism that affect all kidney compartments and involve numerous cellular processes including increased oxidative and nitrosative stress, DNA damage, mitochondrial dysfunction, caspases activation, necrosis, apoptosis and inflammation (Perše and Večerić-Haler 2018).

This stepwise process is initiated by the induction of tubular toxicity and cell death by apoptosis or necrosis, followed by the induction of renal vasoconstriction and then glomerular injury of different compartments including capillaries, basement membrane, podocytes, mesangial cell, and parietal cells and finally interstitial injury (Pabla and Dong 2008).

Clinically, this process is manifested by a reduction of glomerular filtration rate, an increase of serum creatinine, as well as a reduction in serum electrolytes levels notably magnesium and potassium. Finally, the long-term effect of cisplatin may trigger a series of adverse reactions leading to acute renal failure (Oh et al. 2014).

Cannabis is a genus of flowering plants that belongs to the Cannabaceae family in which *Cannabis sativa (C. sativa), Cannabis indica (C.indica), and Cannabis ruderalis (C. ruderalis)*are the most common species recognized worldwide. According to Balant et al. (2024) cannabis phylogenetic and taxonomic studies showed that cannabis can be considered as a single species originating from “Cannabis sativa”. Also, C. sativa split into three group (E Asia, Paleotropis, and Boreal) which can can further subdivided into subgroups. Accordingly, cannabis of the Paleotropis group split into three main subgroups: C & S China and Himalaya, Iranian Plateaus, and Indoafrica. Cannabis from the Boreal group can be subdivided into two subgroups: Eurosiberia and W Mongolia and Caucasus and Mediterranean (Balant et al. 2024).

This aromatic and annual herb has been widely cultivated throughout history and used for its medicinal and industrial purposes (Hartsel et al. 2016). It possesses numerous therapeutic properties naming diuretic, anti-epileptic, anti-emetic, and anti-inflammatory (Ethan 2006, Lozano 1997, Nahas 1982, and Voeks 2014).

In Lebanon, the major cannabis strains found are *C. sativa* and *C. indica.*Recently, a draft law legalizing the cultivation of cannabis for medical and industrial purposes was issued by the Lebanese parliament (University 2020).

Cannabis plant contains different phytochemicals including cannabinoids, terpenes, and phenolic compounds (Andre et al. 2016). Several factors may affect the chemical composition of cannabis strains including age, harvest time, tissue-type, humidity, nutrition, and light levels (Keller et al. 2001, Turner et al. 2017). Tetrahydrocannabinol (THC) and cannabidiol (CBD) are the most important cannabinoids that have been thoroughly studied (Hanuš et al. 2016, Pellati 2018). Accordingly, the psychoactive cannabinoid THC has been identified as a potential drug in the treatment of pain, cancer, multiple sclerosis, and neurodegenerative disorders (Koppel et al. 2014). On the other hand, the non-psychoactive cannabinoid CBD possesses wider pharmacological activities such as anti-inflammatory, antioxidant, antimicrobial, neuroprotective, anxiolytic, and anticonvulsant (Appendino et al. 2011, Campos et al. 2016, and Sangiovanni 2019). Furthermore, the combination of THC and CBD enhances their therapeutic effects when compared to each of the cannabinoids alone (Russo and Guy 2006, and Russo 2011).

To the best of our knowledge, limited studies have been reported about the Lebanese cannabis (McDonald and Gough 1984, Ohlsson et al. 1971, and Shebaby 2021). Moreover, the majority of the previous studies (Baek et al. 2003, Park et al. 2022) focused on the protective effect of cannabis on renal tubular proximal cells in cisplatin-induced nephrotoxicity models, while its effect on podocytes is still unknown. Therefore, the current aimed to study the protective effect of Lebanese Cannabis oil extract in mouse model of cisplatin-induced kidney injury and in vitro using immortalized rat podocytes.

## Materials and methods

### Plant collection and oil extraction

Dried samples of Lebanese cannabis strain were provided through Drug Enforcement Office. Plant extract was prepared as previously described (Shebaby 2021). Briefly, a sample of 10g of air-dried cannabis flower was extracted with ethanol for 48h. The extract was filtered and concentrated at 45ºC under reduced pressure to yield 1.17g of cannabis oil extract (COE).

### Cell cultures

Conditionally immortalized rat glomerular epithelial cells (podocytes) culture kindly provided by.

Dr. Assaad Eid (American University of Beirut) was cultured as previously published (El Zein et al. 2019). Briefly, Podocyte cells cultures propagation was in RPMI media + 10% Fetal Bovine Serum (FBS) and Penicillin–Streptomycin (100U/ml penicillin, 100μg/ml streptomycin). At confluency cells were detached by trypsinization with 1X Trypsin and centrifuged at 1000 rpm for 5 min at 20°C, then re-suspended in fresh complete media. The resuspended cultures were then transferred into new dishes. Induction of growth arrest and differentiation is induced by incubating semi-confluent podocytes without insulin. Accordingly, cell morphology changed from cobblestone to arborized appearance following few days of culture.

### Cell viability assay

Podocyte cells were seeded in 96-well plates (8000 cells/ well). The following day, the cells were incubated in serum free medium composed of 0.1% FBS. After overnight starvation, cells were treated with cisplatin (30µM) in presence or absence of COE (0.5, 1 and 2µg/ml) for 24h. The viability of the cells was assessed using the Cell Titer 96 AQueous Non-Radioactive Cell Proliferation Assay Kit (Promega, USA) which is a colorimetric method based on a reaction of mitochondrial dehydrogenase with 3-(4,5-dimethylthiazol-2-yl)-5-(3-carboxylmethoxyphenyl)-2-(4-sulfophenyl)-2H-tetrazolium inner salt (MTS) as the reagent. Briefly, 20 µL of reagent solution was added to each well of the 96-well plate. After incubation at 37ºC in humidified 5% CO_2_ for 1h, absorbance was read at 490 nm using Eliza microplate reader. Percentage of cell proliferation was determined using the following formula:

$$\frac{\text{Absorbance }{\text{test}}\text{ well}}{\text{Absorbance control well}}\text{x}100$$

### Wound healing assay

Podocyte cells were counted using hemocytometer and plated in 12 well-plates at the density 1 × 10^4^/well. The plates were incubated overnight under growth conditions and allowed for cell recovery and exponential growth. After overnight incubation, the cells were subjected to the serum starvation in 0.1% FBS RPMI for 24 h.

Mechanical scratch representing wound was created in the near confluent monolayer of cells by gently scraping with sterile 200μL micropipette tip. The cells were then rinsed with serum free RPMI and treated with 5μM Cisplatin in the presence or absence of 1µg/ml COE.

Scratch width was photographed at two time points (0 and 24h) from three fields of view with the 10X objective using light microscopy. The experiments were performed in triplicate. Using the Image J software, the cell-free wound surface was measured between the wound edges, averaged between the fields of views and triplicates and the percentage of wound closure was calculated according to the following equation:

$$\frac{(A0-At)}{A0}x100$$

*A*_*0*_ is the initial wound area, *A*_*t*_ is the wound area after n hours of the initial scratch, both in μm^2^.

### Western blot

Podocyte cells were counted using hemocytometer and plated in 6-well plates at the density 25 × 10^4^/well. The plates were incubated overnight under growth conditions and allowed for cell recovery and exponential growth. After overnight incubation, the cells were subjected to the serum starvation in 0.1% FBS RPMI for 24h. Following overnight starvation, the cells were treated with Cisplatin 30µM in presence or absence of 1µg/ml of COE for 24h.

A total of 10^6^ cells for each treatment were pelleted and lysed in RIPA lysis buffer supplemented with protease inhibitor cocktail (Roche). Cell lysates were centrifuged at 12000 g, 4 °C for 15 min and protein concentration was measured using Bradford assay kit (Thermo Fisher). Proteins were separated by SDS–PAGE in reducing conditions and transferred to nitrocellulose membrane. After saturation with 5% milk, the membrane was incubated overnight at 4 °C with the appropriate primary antibody: anti- Bax (1:1000), anti-BCL-2 (1:1000), anti-procaspase-3 (1:1000), anti-cleaved caspase-3 (1:1000), anti-Cox2 (1:1000) and anti-PARP (1:1000). After overnight incubation, the membranes were washed then incubated with (HRP) conjugated secondary antibody for 1 h at room temperature and revealed using the ECL substrate. Monoclonal anti-GAPDH antibody (1/1000; Cell signaling) was used for protein loading control. Densitometric analysis was performed using ImageJ.

### In vivomodel and experimental design

All in vivo experiments were performed on six to eight weeks old black male mice bred in the animal facility at the Lebanese American University. Mice were housed under optimum conditions of temperature (22 ± 2 ◦C) and humidity (50 ± 5%) with an alternating cycle (12h) of light and dark. Animals were supplied with standard laboratory chow diet and water. All experimental protocols were approved by the Lebanese American University Animal Care and Use Committee (ACUC). Mice were divided into six Groups, each group containing 5 mice where mice were injected for three days before sacrifice. For cisplatin treatment, mice were injected with a single dose of cisplatin (20mg/kg, i.p.), while control animals were injected with a comparable volume of PBS 1X. To test the effect of COE, different doses of COE (2.5, 5 and 20 mg/kg, i.p.) dissolved in in a mixture of Ethanol: Tween80: PBS (1:1:18) were administered for three consecutive days before cisplatin treatment. Animals were euthanized 72 h after cisplatin treatment to collect blood samples.

### Serum analysis

Blood samples were collected immediately after euthanasia of the animals. EDTA was then added to prevent clotting of the samples in a ratio of 7μL for each 1 ml of blood collected. Serum was separated using centrifugation at 3000 rpm for 15 min at 4ºC temperature. The concentrations of the renal function biological parameters including serum creatinine and urea were analyzed.

### Urine analysis

Urine samples collected from all mice Groups were diluted in 2X Laemmli Sample Buffer containing 10% β-mercaptoethanol. Equal volumes of each sample were then loaded and resolved under reducing conditions by SDS-PAGE using 4% stacking gel, 10% separating gel and 1X running buffer (0.3% Tris Base, 1.4% glycine, 20% SDS, pH = 8.3) at 90 V for 30 min and then at 120 V for 2 h. After migration, the gel was stained by Coomassie Brillant Blue for two hours and then distained by distilled water overnight. The bands were visualized on Chemidoc bioimaging system.

### Estimated glomerular filtration rate calculation in mice

Estimated glomerular filtration rate (eGFR) was calculated using the following Eqs. (Besseling et al. 2021):

- Plasma creatinine < 52μmol/L: eGFR = 880* W 0.695 * C -0.660 * U -0.391
- Plasma creatinine ≥ 52μmol /L: eGFR = 5862* W 0.695 * C -1.150 * U -0.391

Where:

- eGFR: Estimated GFR (μL/min)
- W: Weight (g)
- C: Creatinine concentration (μmol/L)
- U: Urea (mmol/L)
- 52 μmol/L = 0.937mg/dL

### Tissue preparation

Kidneys were removed from mice and homogenized (10% w/v) in ice cold lysis buffer ( 50 mM Tris–HCl adjusted to a pH = 7.4 (0.5 ml of 1 M), 1% Triton-X, 0.2% Sodium deoxycolate, 1 mM disodium EDTA, 0.2% SDS) using a homogenizer until the pieces are fully homogenized and a homogenous solution is obtained. The mixture was then centrifuged at 4000 × *g* for 5 min at 4 °C. Total protein concentrations were then determined using Bradford assay kit (ThermoFisher). Proteins were then separated by SDS–PAGE in reducing conditions as previously described in western blot section.

### Statistical analysis

Data analysis was expressed as mean ± standard error of mean (SEM). For two group comparison, unpaired t-test was used. Differences between multiple groups were evaluated using one way ANOVA followed by Bonferroni post-hoc test for multiple comparisons. The significance level was accepted with a p value < 0.05 (*), < 0.01 (**), 0.001(***) and < 0.0001(****). Statistical analysis was performed using GraphPad Prism 8.4.

## Results

### COE prevented podocytes apoptosis induced by cisplatin

**Fig. 1:**
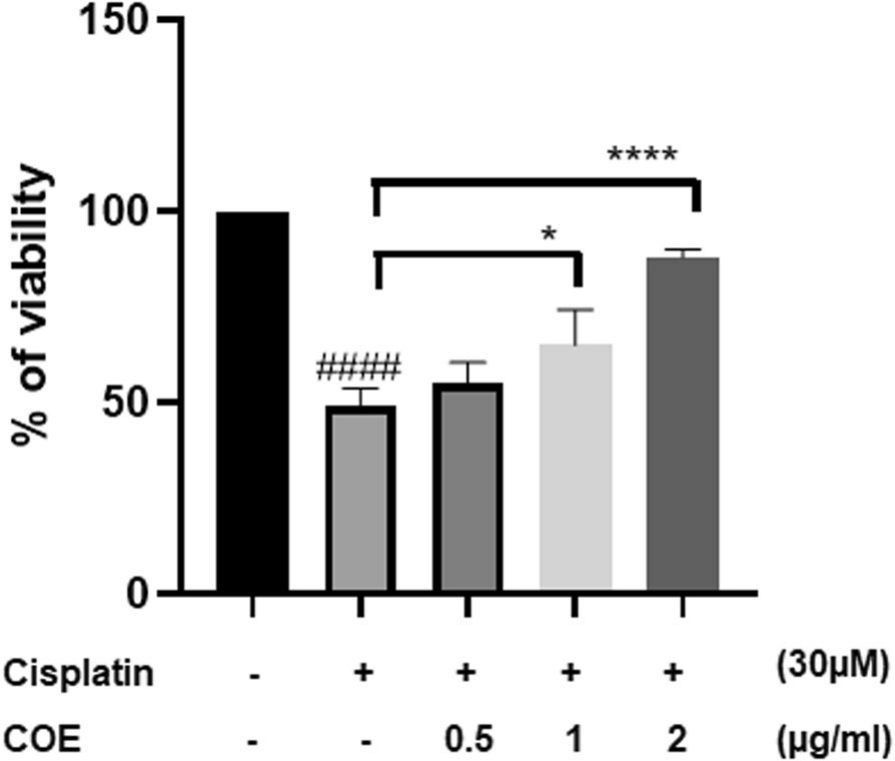
Cannabis Oil Extract protects podocyte cells from cisplatin-induced cytotoxicity. Rat podocytes were treated with 30 μM cisplatin in the absence or presence of Cannabis Oil Extract (COE) at the indicated concentrations for 24 h. Cell viability was evaluated via Cell Titer 96 Aqueous Non-Radioactive Cell Proliferation Assay. Data are expressed as mean ± SD (*n* = 3). Differences between groups were evaluated using one-way ANOVA followed by Bonferroni’s multiple comparison test. #### P < 0.0001 vs control, *P < 0.05, ****P < 0.0001 significantly different from the cisplatin-only treated group

To test whether COE protects podocyte cells from cisplatin-induced cytotoxicity, cells were treated with 30μM cisplatin and 0.5, 1 and 2μg/ml COE for 24h respectively. Accordingly, treatment of podocytes with 30μM cisplatin for 24h showed 50% cell viability reduction, while COE alone did not affect cell viability when compared to control non-treated cells (Supplementary Fig. 1A and B). Interestingly, cell viability assay showed that COE attenuated cisplatin-induced cytotoxicity in a concentration-dependent manner in podocyte cells (Fig. 1). While cells treated with cisplatin showed 50% reduction in cell viability, coincubation of podocytes with cisplatin + 2μg/ml COE showed an increase in cells viability up to 87% when compared to cisplatin-treated cells.

**Fig. 2:**
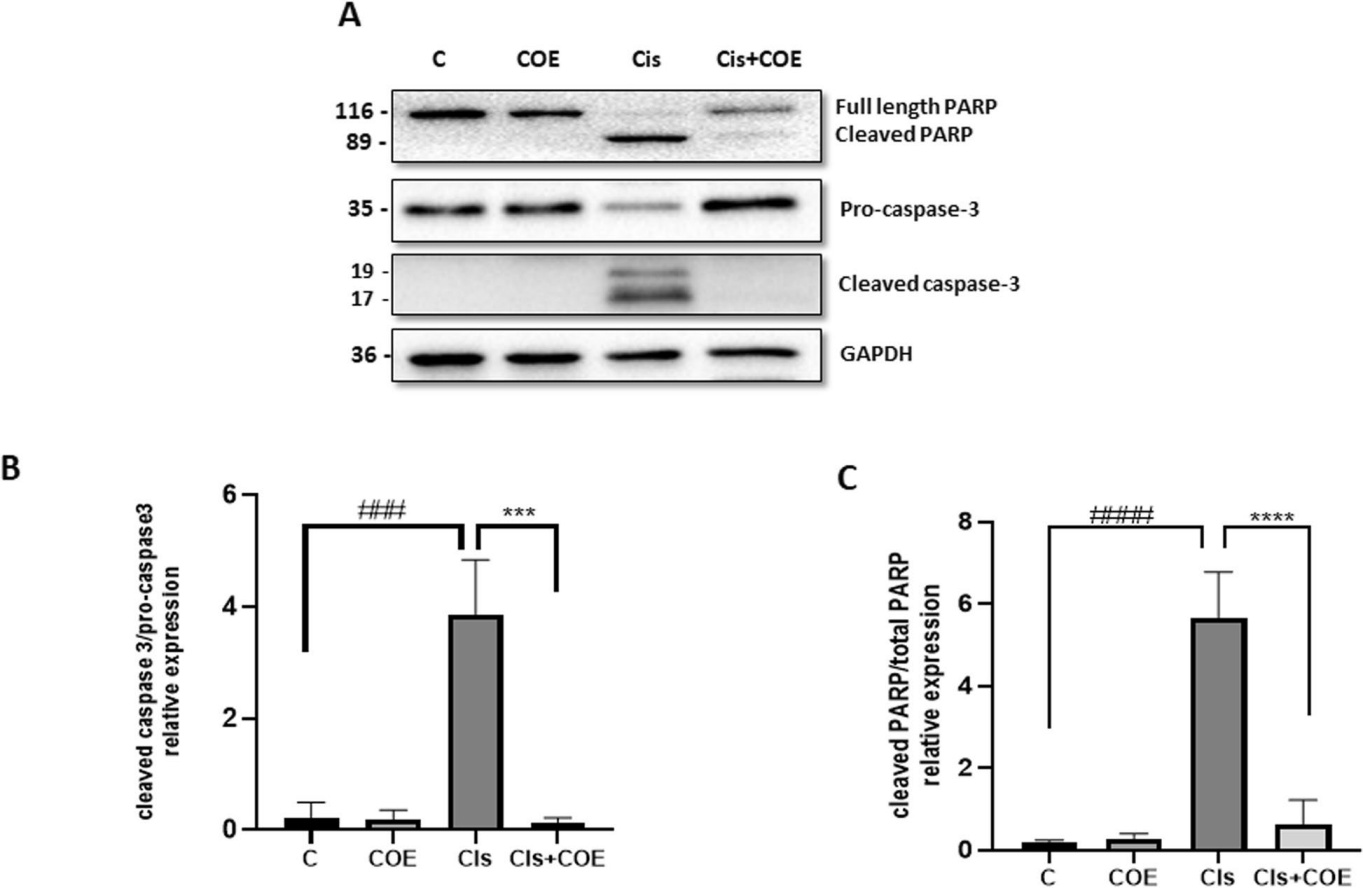
Cannabis Oil Extract protects podocyte cells from cisplatin by inhibiting apoptosis. **A** Representative immunoblot analysis. **B** and **C** Densitometric analysis of immunoblots to estimate the relative abundance of indicated protein as normalized to that of GAPDH. Data are expressed as mean ± SD (n = 3). Differences between groups were evaluated using one-way ANOVA followed by Bonferroni’s multiple comparison test. ### *P* < 0.001 vs control, #### *P* < 0.0001 vs control, ***P < 0.001 significantly different from the cisplatin-only treated group

Furthermore, western blot analysis showed that COE treatment decreased the expression of various apoptosis markers including cleaved caspase-3/pro-caspase-3 ratio (Fig. 2A and B) and cleaved PARP/total PARP (Fig. 2A and C) when compared to cisplatin-only treated cells (Fig. 2). These results suggest that COE protects podocyte cells from cisplatin-induced cytotoxicity by inhibiting apoptosis.

### COE partially reversed migration inhibition in cisplatin-treated podocytes

**Fig. 3:**
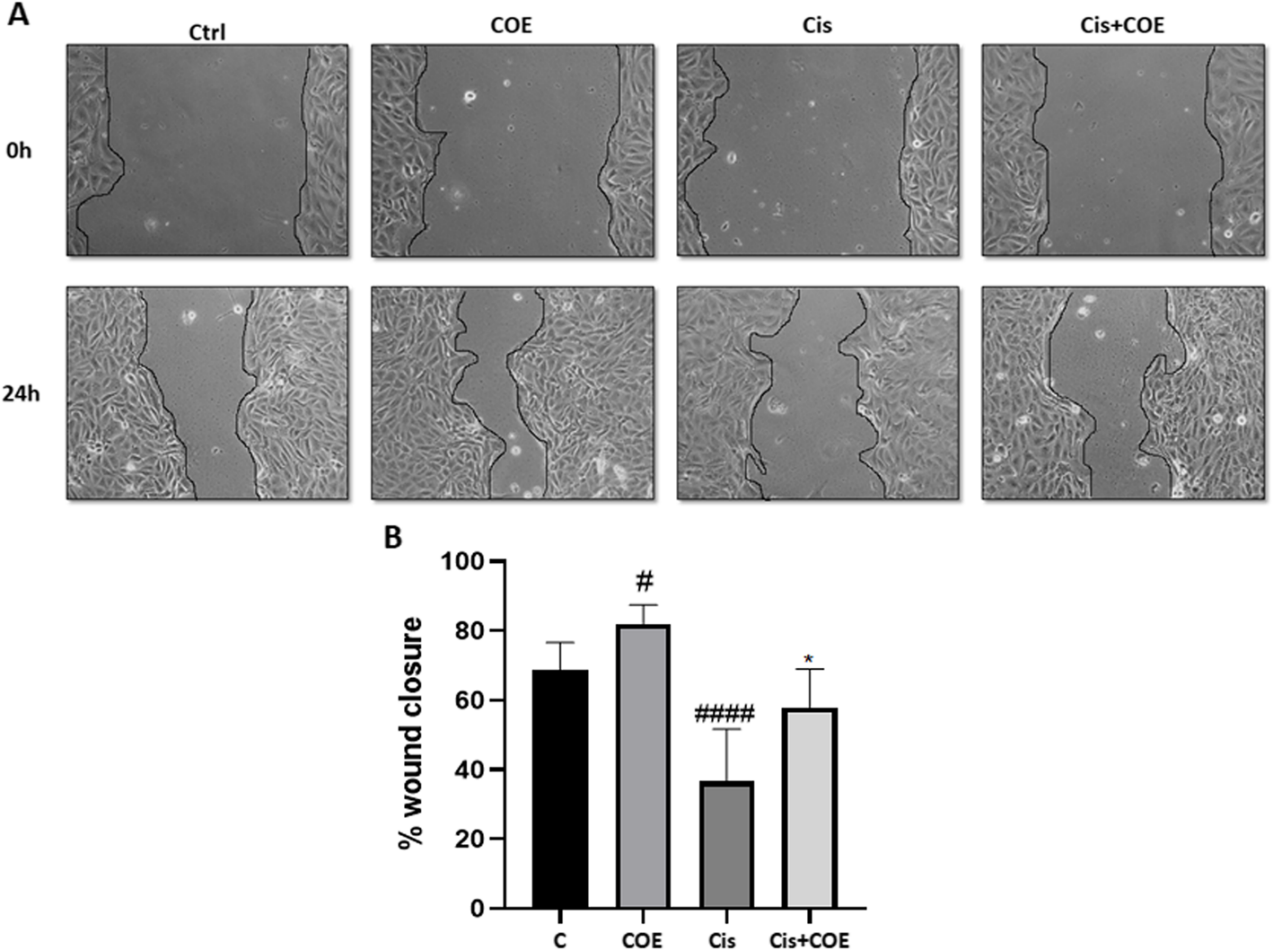
Podocytes cell migration. **A** representative pictures show the migration of podocyte cells after induction of a scratch representing wound. All the pictures were caught immediately after the scratch was induced (at zero hour) and after 24 h of incision. Podocytes on the pictures were cultured in different conditions as indicated. Pictures are taken at 10 times magnification. **B** The relative change in cell-free gap surfaces was measured at different time points after creation of the wounding scratch and expressed as fold change over zero time. Results are expressed as mean ± SEM of three independent experiments performed in triplicate. Statistictical analysis was performed using two-way ANOVA followed by Bonferroni’s multiple comparison test. # P < 0.05 vs. control, ###P < 0.001 vs. control, *P < 0.05 vs. cisplatin

In vitro scratch assay was performed in order to measure the rate of podocyte cells migration in the presence of 5μM cisplatin and or 1μg/ml COE. As illustrated in Fig. 3A and B, COE accelerated wound closure (82 ± 5.47%) as compared to control non-treated cells (68 ± 7.9%). As expected, cisplatin significantly slowed down the closure of the cell free gap after 24h (36.6 ± 15%). Co-treatment of podocytes with cisplatin and COE partially reversed the inhibitory effect of cisplatin and restored wound closure (57.96 ± 11%) after 24h when compared to cells treated with cisplatin alone.

### Protective Effect of COE against cisplatin-induced renal toxicity

To investigate the effect of COE on cisplatin-induced renal toxicity in animal models, levels of serum urea and creatinine were measured 72 h after cisplatin administration.

**Fig. 4:**
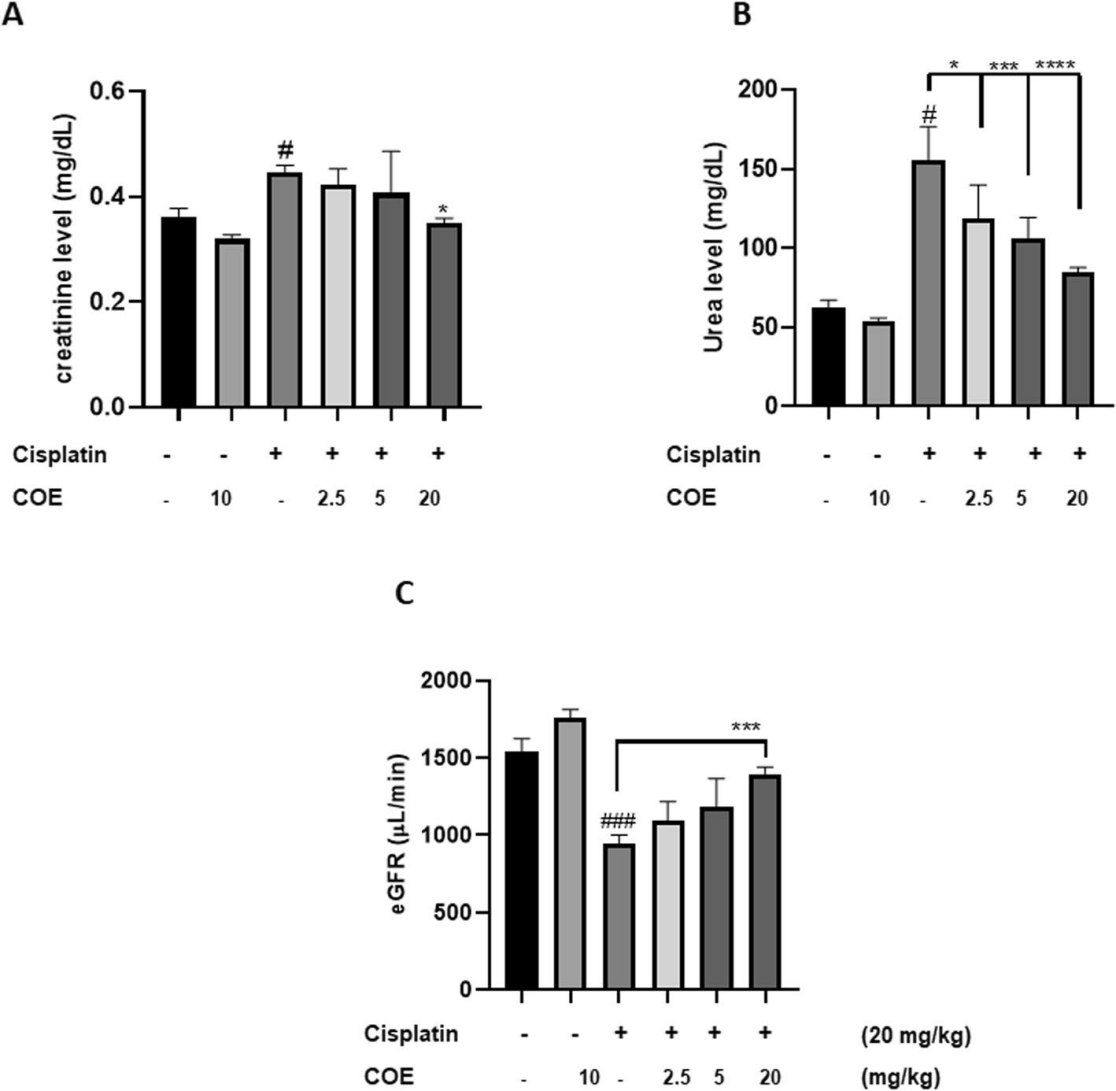
Average serum creatinine levels (mg/dL) (**A**) average serum urea levels (mg/dL) (**B**) and Estimated GFR (μL/min) (**C**) in different Groups of mice in *C. sativa* experiment. COE was injected i.p. once for three days. On day one, COE was injected 90 min before cisplatin injection (20 mg/kg, i.p.). Serum urea and creatinine levels were measured 72 h after cisplatin injection. Each column represents the mean ± SEM of five animals. Statistictical analysis was performed using two-way ANOVA followed by Bonferroni’s multiple comparison test. # *P* < 0.05 vs. control, ### *P* < 0.001 vs. control, * *P* < 0.05, ***P < 0.001, ****P < 0.0001 significantly different from the cisplatin-only treated group

As shown in Fig. 4, intraperitoneal injection of cisplatin produced an elevation of both serum creatinine and urea levels in mice when compared to normal control groups. Survival rate among the animals treated with cisplatin was 100%.

Administration of COE at 20 mg/kg for three days after intraperitoneal cisplatin (20 mg/kg) injection in mice significantly decreased both serum creatinine and urea levels (Fig. 4A and B). However, administration of lower doses of COE (2.5 or 5 mg/kg) caused significant decrease in serum urea (Fig. 4B) levels but were without effect on serum creatinine (Fig. 4A).

### Attenuation of cisplatin-induced decrease in GFR

Mice treated with cisplatin alone at a dose of 20mg/kg showed a marked decrease in eGFR when compared to vehicle-treated group (Fig. 4C). Injection of COE (20mg/kg) once daily for 3 consecutive days after cisplatin injection caused significant increase in eGFR.

### Attenuation of cisplatin-induced increased albumin excretion in urine

**Fig. 5:**
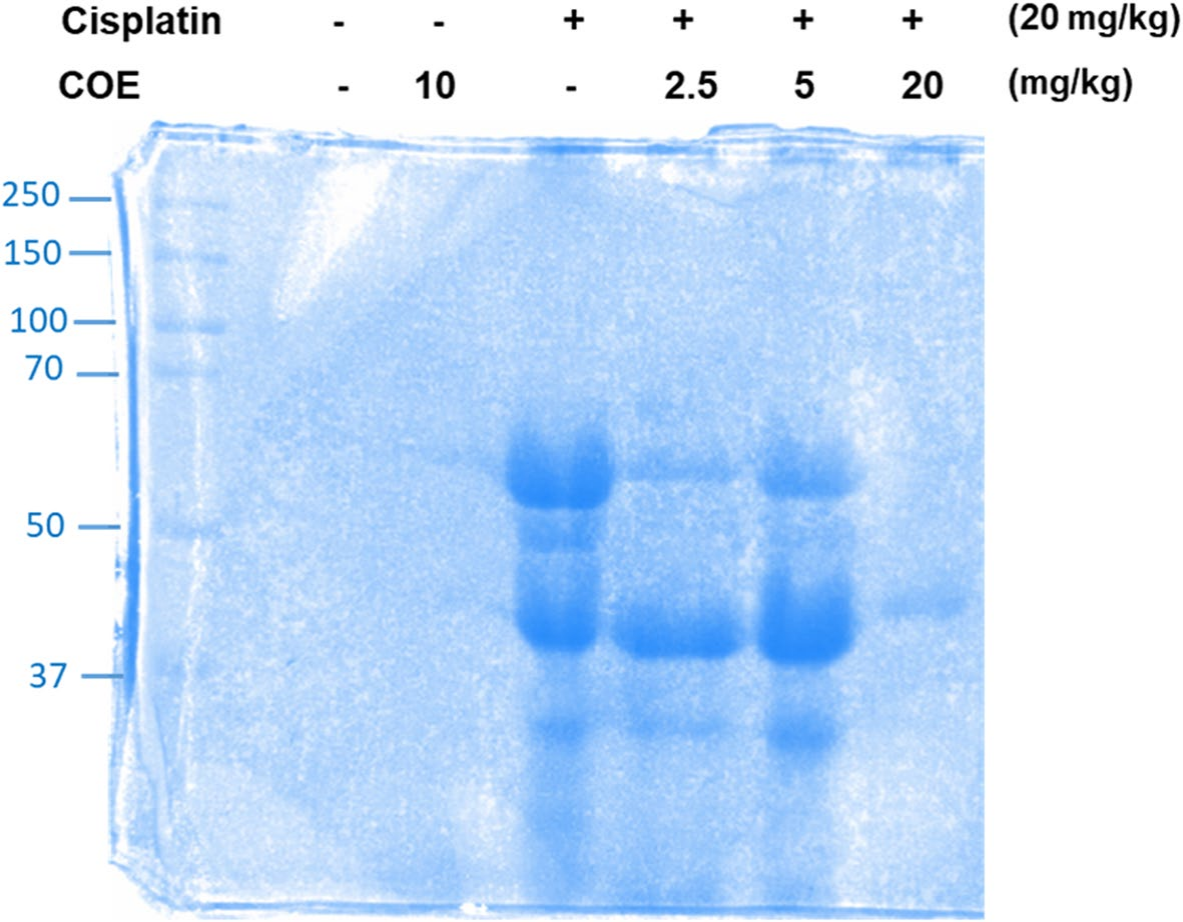
Cannabis oil extract reduces albumin in urine of cisplatin treated mice. SDS-PAGE study for albumin detection in urine samples of mice.The samples were run on a Bio-Rad protein electrophoresis equipment and staining was performed with Coomassie blue. The bands at 65 KDa represent albumin

Urine samples were collected from control, cisplatin and COE treated groups were analyzed by SDS-PAGE for proteinuria (Fig. 5). Both control and COE groups showed very faint and barely detectable protein bands at 65KDa indicating a minimal presence of albumin in urine. On the contrary, cisplatin-treated mice showed a much larger and more intensely stained albumin bands indicating a higher presence of albumin protein in urine. Interestingly, treatment with COE (2.5, 5 and 20mg/kg) showed marked decrease in the intensity of albumin bands when compared to cisplatin-treated mice group indicating that COE treatment reduced the leakage of protein in urine induced by cisplatin.

### COE attenuated cisplatin-induced COX-2 protein expression and apoptosis protein markers in mice kidneys

**Fig. 6:**
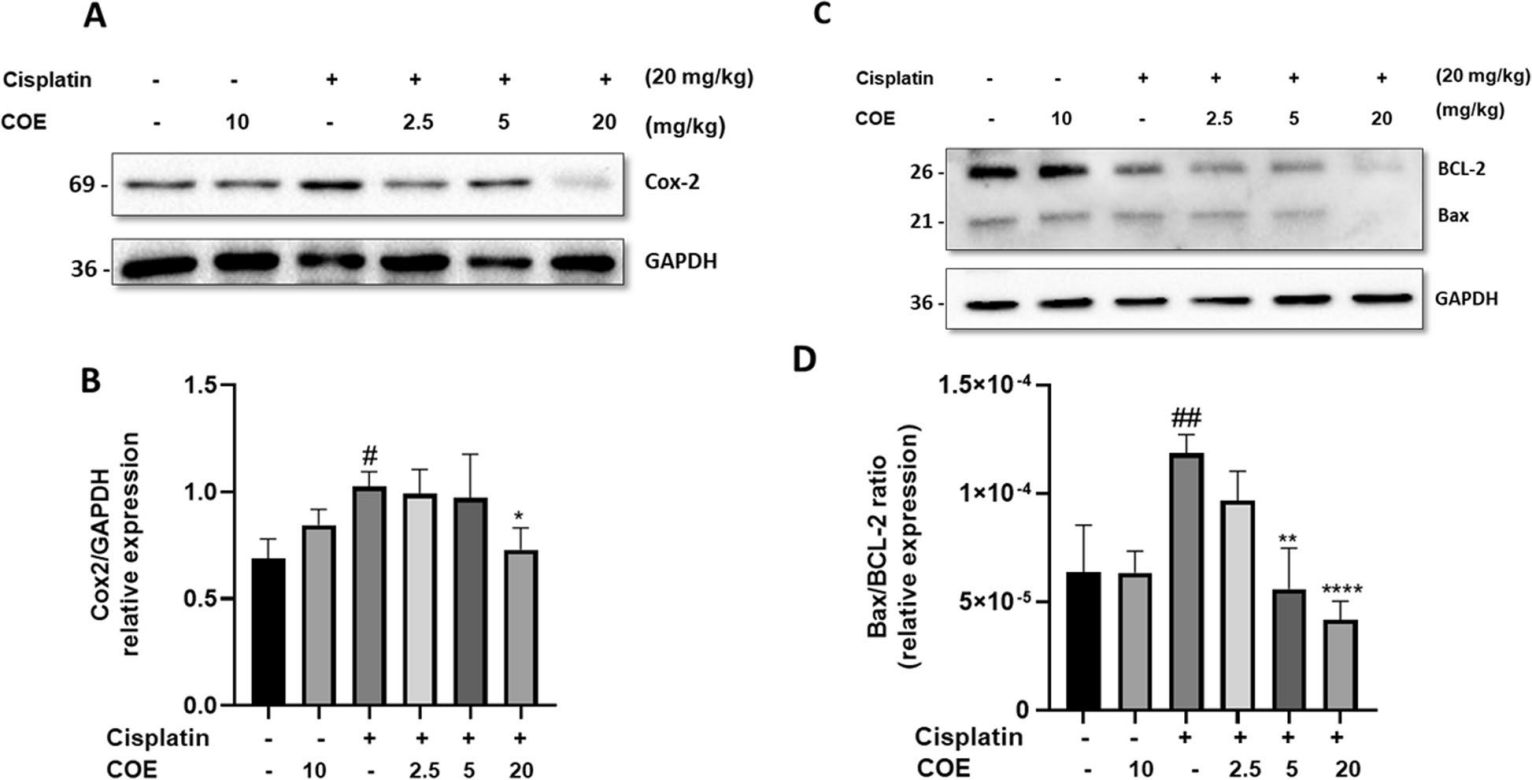
Cannabis Oil Extract protects kidneys from cisplatin by inhibiting apoptosis and inflammation. **A**, **C** Representative immunoblot analysis. **B**, **D** Densitometric analysis of immunoblots to estimate the relative abundance of indicated protein as normalized to that of GAPDH. Data are expressed as mean ± SD (*n* = 3). Differences between groups were evaluated using one-way ANOVA followed by Bonferroni’s multiple comparison test. #*P* < 0.01 vs control, ##*P* < 0.001 vs control, **P < 0.001, ****P < 0.0001 significantly different from the cisplatin-only treated group

Mice injected with cisplatin showed significantly increased COX-2 protein levels in their kidney (Fig. 6A and B). Addition of COE to the treatment regimen reduced COX-2 protein levels with a maximum inhibition observed in mice treated with a COE dose of 20mg/Kg. Furthermore, COE treatment decreased Bax/BCL-2 protein ratio when compared to cisplatin-treated mice group (Fig. 6C and D). These results suggest that COE protects kidneys from cisplatin-induced cytotoxicity by mostly inhibiting inflammation and apoptosis.

## Discussion

Cisplatin is one of the most widely chemotherapeutic drug used for the treatment of many solid tumors. Cisplatin therapy is often associated with various adverse effects including vomiting, ototoxicity, myelosuppression, allergic reactions and nephrotoxicity, being the major dose-limiting side effect with a prevalence of 30% in patients undergoing cisplatin therapy (Sastry et al. 2005). Therefore, it is imperative to develop strategies to protect the kidneys from the toxic effects without lowering cisplatin tumoricidal activity.

After administration, cisplatin is actively transported into the nephrons and more precisely the proximal tubular cells of the inner cortex and the outer medulla, which absorb the highest concentrations of the drug. At these sites, cisplatin induces nephrotoxicity and cell loss in a complex process that involves increased oxidative, and nitrosative stress, DNA damage, mitochondrial dysfunction, caspases activation, necrosis, apoptosis as well as inflammation (Perše et al. 2018). Thus, agents that may protect renal tissues from oxidative stress, inflammatory and show cytoprotective properties, could be used in conjunction with chemotherapy treatments to lessen the nephrotoxic and damaging effects of drugs like cisplatin (Husain et al. 1998). Previous experiments in our laboratory showed that COE demonstrated potent in vitro and in vivo anti-inflammatory activities (Shebaby 2021).

In this study, the nephroprotective effects of Lebanese *C. sativa*, in vitro and in animal model were investigated.

Although, tubular cell damage and death is the most common histopathological feature of cisplatin nephrotoxicity, chronic exposure to cisplatin is associated with significant tubular atrophy and interstitial fibrosis and relative glomerular sparing (Cornelison et al. 1993).

The majority of the in vitro experiments of cisplatin-induced cytotoxicity use proximal tubular cells as a model, and to the best of our knowledge this is the first model that evaluated the effect of cannabis on kidney podocytes. Podocytes are defined as highly specialized glomerular epithelial cells that are play pivotal role in maintaining the integrity and the function of glomerular filtration barrier and the onset of proteinuria (Gao 2019).

We started by evaluating the effect of cisplatin on podocytes viability. Our results showed that podocytes cell viability was reduced by 50% upon treatment with 30µM cisplatin after 24h. These results are in agreement with various in vitro studies that demonstrated similar cisplatin cytotoxicity on proximal tubular cells (Baek et al. 2003; , Park et al. 2022; , Lieberthal 1996).

Moreover, in a model of acute kidney injury using human kidney organoids, podocyte cells were found to be the least damaged by cisplatin treatment when compared to proximal tubule cells, distal tubule cells and interstitial cells (Digby et al. 2020). However, in an in vivo model of guinea pigs, cisplatin exerted damaging effects on both the tubular region of the kidney and its glomerular components including glomerular capillaries, basement membrane, epithelial podocytes, mesangial cells, and parietal cells of Bowman’s capsule (Kohn et al. 2002). All these results demonstrated that podocytes could be used as a model to investigate cisplatin-induced nephrotoxicity in vitro.

We next moved to investigate the effect of COE in this model. Our in vitro studies showed that COE protected podocyte cells from cisplatin-induced cytotoxicity in a concentration-dependent manner. These results are in agreement with a recent study (Marzęda 2022) that demonstrated an antagonistic effect on cell viability of CBD and cisplatin in three different malignant melanoma cell lines (SK‐MEL28, A375 and FM55P). Deng et *al. *(2017) also obtained similar results in glioblastoma cell lines, where the co-administration of low concentrations of CBD antagonized cisplatin cell killing activity in all studied cell lines (Deng et al. 2017).

Mechanistically, in vitro results showed that COE inhibits the expression of various apoptosis markers (cleaved caspase-3 and cleaved PARP) that were induced upon cisplatin treatment. Induction of apoptosis and more specifically the intrinsic pathway is a well-characterized mechanism of cisplatin-induced cytotoxicity (Baek et al. 2003, Park et al. 2022, Lieberthal 1996). However, the role of COE on the induction or inhibition of apoptosis is governed by several experimental conditions including but not limited to the cell line (malignant or non-malignant), the extraction method and phyto-substances in case of extract and the dose-dependency with lower concentrations being more cytostatic than inducing apoptosis as in our model (Nahler 2022, Rožanc 2021).

As for the in vitro wound-healing assay, our results demonstrated that COE stimulated cell migration and restored the migratory capacity of podocytes that was lost upon treatment with cisplatin. Similarly, to the effect of COE on the expression of apoptotic markers, the effect of COE on cell migration is also governed by the same aforementioned factors. Accordingly, Cannabis-derived compounds cannabichromene and D9-Tetrahydrocannabinol and the CBD-THC combination inhibited urothelial cell carcinoma and multiple myeloma cell migration respectively (Anis et al. 2021, Nabissi et al. 2016). On the contrary, Cannabidiol significantly improved wound healing in human brain endothelial cells (Anis et al. 2021, Nabissi et al. 2016) and fibroblasts (Gerasymchuk et al. 2022, Luo et al. 2019).

The inhibitory effect of cisplatin on podocytes cell migration is in agreement with a recent study in which podocytes were treated with high glucose condition to model diabetic nephropathy. Similarly to high glucose, cisplatin-treated podocytes demonstrated a damage to the junctions between the cells and subsequent loss of their cellular integrity reminiscent of the denuded zone created in in vitro scratch assay (Bejoy et al. 2022).

Besides podocytes, cisplatin as anti-cancer drug was found to inhibit cell migration of several cancer cell lines including but not limited to ovarian, head and neck and prostate cancer (Mortensen et al. 2020, Raudenska et al. 2019, Wang et al. 2018).

The in vitro results were further verified in a murine model of cisplatin-induced nephrotoxicity. In order to assess kidney function, serum creatinine and urea, eGFR and proteinuria were evaluated. Creatinine clearance, which reflects the glomerular filtration rate (GFR), may be calculated using blood creatinine levels as a marker of renal function (Gounden et al. 2023). In the present study, cisplatin treatment caused significant increase in functional nephrotoxicity markers such as serum creatinine and urea levels, indicating intrinsic acute renal damage. Treatment with various doses of COE significantly decreased serum urea, and COE at a dose of 20 mg/kg was able to significantly reduce serum creatinine and improve eGFR.

We further assessed the presence of proteins in urine. Gel electrophoresis results revealed a marked increase in protein in the collected urine after cisplatin treatment when compared to the control. However, in the COE treated groups the protein level was markedly decreased compared to the cisplatin, group thus reflecting the reno-protective effect of COE. In fact, small amounts of albumin can also be due to stress and diet; however, increased levels of albumin indicate kidney damage (Gounden 2023).

The reduction of glomerular filtration rate as well as the increased plasma creatinine and urea levels and urine albuminuria and proteinuria have been widely reported in various cisplatin rodent model that has been now recognized as a simple and reproducible model with high clinical relevance (Perše et al. 2018).

Our results are in line with other published reports evaluating the nephroprotective role of CBD and Cannabigerols (CBGA) in mice. The first study showed that both 5 and 10mg/kg doses of CBD significantly decreased serum creatinine and urea (Pan et al. 2009). In the second study, CBGA, CBD and a combination of CBGA + CBD strongly reduced serum creatinine and urea in cisplatin-treated mice (Suzuki et al. 2023). The latter indicates that COE can provide significant protection against cisplatin-induced nephrotoxicity mainly on the tubular level, which is the site of creatinine and urea excretion.

Cannabinoids exert their effects by interacting with the receptors of the endocannabinoid system. In fact, the potential role of EC system in treating various kidney diseases has been an emerging area of research, specifically in the context of cannabinoid receptors (Nettekoven et al. 2016). The two types of receptors (*CB1* and *CB2*) that are activated by the pharmacologically active ingredients of cannabis are found in numerous tissues, including the kidneys (Park et al. 2017). Cannabidiol, which acts as an antagonist/ inverse agonist by weakly binding to both*CB1* and *CB2*receptors, was found to partially prevent renal tubular injury after bilateral renal ischemia/reperfusion (Nettekoven et al. 2016). Another series of studies using cisplatin-induced renal injury yielded similar results where the blocking the*CB1* receptor or activating the *CB2*receptor has been shown to protect against tubular damage by reducing oxidative stress and inflammation in the kidney (Horváth et al. 2012, Mukhopadhyay et al. 2016, Mukhopadhyay et al. 2010). Alterations of cannabinoid receptors have been involved in different renal diseases such as acute kidney injury, chronic kidney disease, and diabetic nephropathy (Park et al. 2017). Hence, targeting the EC system in treating nephrotoxicity may present some therapeutic value. As for the other constituents of COE, it contains several common terpenes, mainly β-caryophyllene (Shebaby 2021). Previous study showed that the natural product, β- caryophyllene, which can act as a full CB2 agonist, could protect the kidney from the harmful effects of cisplatin through decreasing inflammation and oxidative stress (Horváth et al. 2012, Nuutinen 2018). In our cell model, the exact mechanisms by which*CB1* and *CB2* can protect podocytes against cisplatin damaging effect is currently under investigation.

Furthermore, COE is expected to produce more potent effect than using pure compounds alone due to the synergism of its various cannabinoids and terpenes, a phenomenon known as the "entourage effect" (Gallily et al. 2019). Because Lebanese COE contains approximately 59% CBD (Shebaby 2021), the 2.5, 5 and 20mg/kg doses of COE used in our study were nearly equivalent to 1.5, 2.95, and 11.8mg/kg of CBD, respectively. All these COE doses significantly reduced serum urea and were shown to be reno-protective in mice. However, in a similar study done on mice where CBD was used alone, only 5 and 10mg/kg doses significantly decreased serum urea with no significance for 2.5mg/kg dose (Pan et al. 2009). Thus, the fact that low COE doses (corresponding to 1.5 and 2.95 mg/kg doses of CBD) were more effective than similar dose of CBD used alone (2.5 mg/kg) support the “entourage effect” phenomena.

Mechanistically, in addition to the apoptosis markers (Bax/Bcl2 ratio), western blot analysis was used to detect COX-2 expression in the kidneys of mice. COX-2 is an inducible isozyme that is produced in response to inflammatory stimuli (Yu et al. 2017). In our model, COX-2 protein overexpression in kidney tissues of the cisplatin-treated animal group was used as an indicator to describe renal inflammation. COE treatment reduced COX-2 and Bax/Bcl2 ratio expression mainly at a dose of 20 mg/kg dose. These results are consistent with previous findings in which cannabidiol ameliorated ischemia/reperfusion-induced kidney damage by reducing the expression of*cyclooxygenase-2*expression (Fouad et al. 2012). Furthermore, another study have demonstrated that Cannabidiol attenuated cisplatin-induced PARP and caspase-3 activities in mice in the context of apoptosis, as well as reduced the protein expression of*inducible nitric oxide (iNOS)*, *IL1-β* and *TNF-α*mRNA expression in the context of inflammation (Pan et al. 2009). Moreover, Suzuki et *al.*(2023) recently showed both CBGA and CBD suppressed mRNA expression of inflammatory cytokines in cisplatin-induced nephropathy and reduced apoptosis through inhibition of caspase-3 activity (Suzuki et al. 2023). Similar results were found but with another study but using different medication. Accordingly, in a recent study Soliman et al., (2024) showed that cannabidiol oil protected against doxorubicin-induced alterations in renal function and abnormalities in kidney tissues as well as caused reduction of inflammation (Soliman et al. 2024). Finally, further studies are required to examine other strain or components of Lebanese cannabis oil and investigate the signaling pathways and the mechanisms of action underlying their nephroprotective effect against cisplatin toxicity without compromising its anticancer effects.

## Conclusion

In conclusion, our results corroborated previous findings but on kidney podocytes. We strongly suggest that the Lebanese Cannabis oil extract may be of significant therapeutic benefits against the renal complications of cisplatin. Thus, Lebanese COE produces its renoprotective effects partly through activating antiinflammatory and antiapoptoric mechanisms in podocytes.

## Supplementary Information

Additional file 1. Supplementary Figure 1. Effect of Cisplatin and Cannabis Oil Extract (COE) on podocytes cell viability. Cell viability was evaluated using Cell Titer 96 Aqueous Non-Radioactive Cell Proliferation Assay Kit. Data are expressed as mean±SD. Differences between groups were evaluated using one-way ANOVA followed by Bonferroni’s multiple comparison test. *P<0.05, ****P<0.0001 significantly different from control. (A) Podocyte cells were treated with indicated concentrations of cisplatin for 24 h (*n *= 4). (B) Podocyte cells were treated with indicated concentrations of Cannabis oil extract (COE) for 24 h (*n *= 3).

## Data Availability

The datasets used and/or analysed during the current study are available from the corresponding author on reasonable request.

## Abbreviations

AKI: Acute kidney injuries
ATP: P-type copper transporting ATPases
Bax: Bcl-2-associated X protein
Bcl-2: B-cell lymphoma 2
BUN: Blood urea nitrogen BUN
CB: Cannabinoid receptors
CBD: Cannabidiol
CBGA: Cannabigerols
CIN: Cisplatin-induced nephrotoxicity
COE: Cannabis oil extract
COX-2: Cyclooxygenase-2
CTR: Copper transporter
DNA: Deoxyribonucleic acid
EC: Endocannabinoid
eGFR: Estimated glomerular filtration rate
FBS: Fetal Bovine Serum
FDA: Food and Drug Administration
GAPDH: Glyceraldehyde-3- phosphate dehydrogenase
IL-1β: Interleukin-1 beta
iNOS: Inducible nitric oxide
MATE: Multidrug extrusion transporter- 1
mRNA: messanger Ribonucleic acid
MTS: [*3-(4,5-dimethylthiazol-2-yl)-5-(3-carboxymethoxyphenyl)-2-(4-sulfophenyl)-2H-tetrazolium*]
OCT: organic cationtransporter and;
PARP: poly-ADP ribose polymerase
PBS: Phosphate Buffer saline
RPMI: Roswell Park Memorial Institute
SDS: Sodium Dodecyl Sulfate
SDS-PAGE: Sodium dodecyl-sulfate polyacrylamide gel electrophoresis
THC: Tetrahydrocannabinol
TNF-α: Tumour Necrosis Factor alpha
