# Supplementary figures and images for: Lebanese Cannabis sativa L. extract protects from cisplatin-induced nephrotoxicity in mice by inhibiting podocytes apoptosis
Source: J Cannabis Res. 2025 Jan 16;7:3. doi: 10.1186/s42238-025-00260-4 (PMC11737194; doi:10.1186/s42238-025-00260-4)

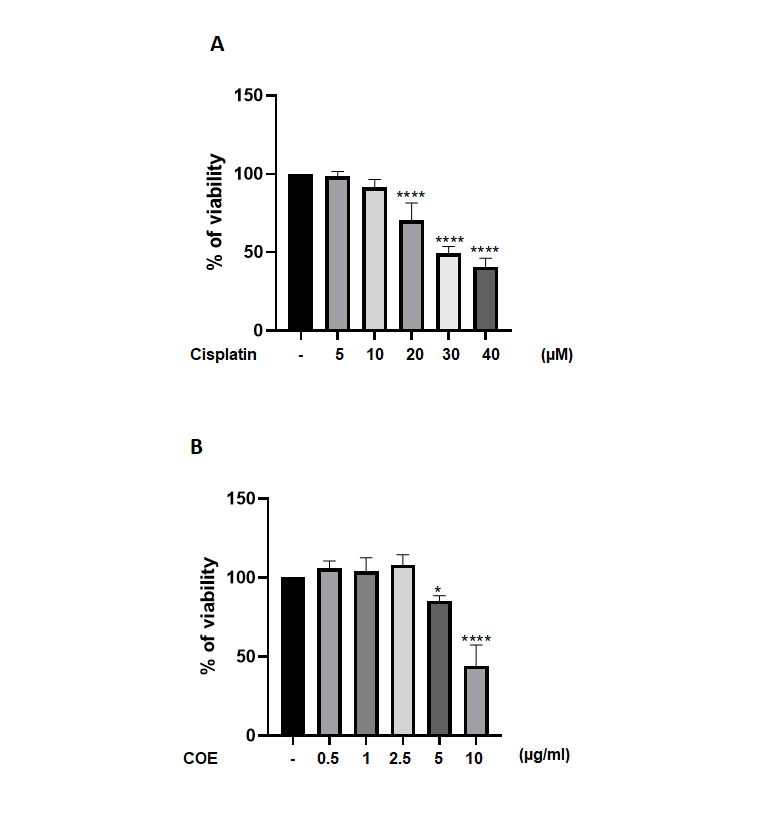

Supplement: Supplementary file 1 — Additional file 1. Supplementary Figure 1. Effect of Cisplatin and Cannabis Oil Extract (COE) on podocytes cell viability. Cell viability was evaluated using Cell Titer 96 Aqueous Non-Radioactive Cell Proliferation Assay Kit. Data are expressed as mean±SD. Differences between groups were evaluated using one-way ANOVA followed by Bonferroni’s multiple comparison test. *P<0.05, ****P<0.0001 significantly different from control. (A) Podocyte cells were treated with indicated concentrations of cisplatin for 24 h (n = 4). (B) Podocyte cells were treated with indicated concentrations of Cannabis oil extract (COE) for 24 h (n = 3). [file 42238_2025_260_MOESM1_ESM.tif]
